# Problems with Bazett QTc correction in paediatric screening of prolonged QTc interval

**DOI:** 10.1186/s12887-020-02460-8

**Published:** 2020-12-14

**Authors:** Irena Andršová, Katerina Hnatkova, Kateřina Helánová, Martina Šišáková, Tomáš Novotný, Petr Kala, Marek Malik

**Affiliations:** 1 Department of Internal Medicine and Cardiology, Faculty of Medicine, University Hospital Brno, Masaryk University, Brno, Czech Republic; 2 National Heart and Lung Institute, Imperial College, ICTEM, Hammersmith Campus, 72 Du Cane Road, Shepherd’s Bush, London, W12 0NN England

**Keywords:** Long QT screening, QTc prolongation in children, Bazett correction, Fridericia correction, Framingham correction

## Abstract

**Background:**

Bazett formula is frequently used in paediatric screening for the long QT syndrome (LQTS) and proposals exist that using standing rather than supine electrocardiograms (ECG) improves the sensitivity of LQTS diagnosis. Nevertheless, compared to adults, children have higher heart rates (especially during postural provocations) and Bazett correction is also known to lead to artificially prolonged QTc values at increased heart rates. This study assessed the incidence of erroneously increased QTc values in normal children without QT abnormalities.

**Methods:**

Continuous 12-lead ECGs were recorded in 332 healthy children (166 girls) aged 10.7 ± 2.6 years while they performed postural manoeuvring consisting of episodes (in the following order) of supine, sitting, standing, supine, standing, sitting, and supine positions, each lasting 10 min. Detailed analyses of QT/RR profiles confirmed the absence of prolonged individually corrected QTc interval in each child. Heart rate and QT intervals were measured in 10-s ECG segments and in each segment, QTc intervals were obtained using Bazett, Fridericia, and Framingham formulas. In each child, the heart rates and QTc values obtained during supine, sitting and standing positions were averaged. QTc durations by the three formulas were classified to < 440 ms, 440–460 ms, 460–480 ms, and > 480 ms.

**Results:**

At supine position, averaged heart rate was 77.5 ± 10.5 beat per minute (bpm) and Bazett, Fridericia and Framingham QTc intervals were 425.3 ± 15.8, 407.8 ± 13.9, and 408.2 ± 13.1 ms, respectively. At sitting and standing, averaged heart rate increased to 90.9 ± 10.1 and 100.9 ± 10.5 bpm, respectively. While Fridericia and Framingham formulas showed only minimal QTc changes, Bazett correction led to QTc increases to 435 ± 15.1 and 444.9 ± 15.9 ms at sitting and standing, respectively. At sitting, Bazett correction identified 51, 4, and 0 children as having the QTc intervals 440–460, 460–480, and > 480 ms, respectively. At sitting, these numbers increased to 118, 11, and 1, while on standing these numbers were 151, 45, and 5, respectively. Irrespective of the postural position, Fridericia and Framingham formulas identified only a small number (< 7) of children with QT interval between 440 and 460 ms and no children with longer QTc.

**Conclusion:**

During screening for LQTS in children, the use of Bazett formula leads to a high number of false positive cases especially if the heart rates are increased (e.g. by postural manoeuvring). The use of Fridericia formula can be recommended to replace the Bazett correction not only for adult but also for paediatric ECGs.

## Background

The fact that the QT interval duration shortens with increasing heart rate has been known practically from the beginning of electrocardiography. This knowledge only extended the observations of the heart rate influence on the duration of cardiac systole that were made more than 150 years ago, well before the first ever electrocardiogram was recoded [1].

Nevertheless, only some 2 decades ago, it was observed that the relationship between the QT interval and the underlying heart rate shows not only substantial inter-subject differences but also considerable intra-subject stability [2, 3]. The implications for clinical assessment of rate corrected QTc interval changes became well recognised [4] both in clinical practice and in the investigations of repolarisation changing properties of novel pharmaceutical compounds. The knowledge that no generic and universal heart rate correction formula can possibly exists that would reasonably correct the QT interval in all subjects led to the practice of using generic formulas (such as Bazett [5], Fridericia [6], Framingham [7], or Hodges [8] formulas) only in cases when the heart rate is not markedly different from baseline conditions, e.g. around 60 beats per minute (bpm) [9]. When QT correction is needed in other situations, individual QT/heart rate profiles need to be studied so that the individual-specific QT adaptation to rate changes can be taken into account.

In children, baseline heart rate is usually substantially higher than 60 bpm [10]. Consequently, the implications of the QT-heart rate individuality are little used in paediatric practice. Despite the knowledge that in adults, Bazett formula leads to artificially prolonged QTc values when applied to recordings of increased heart rate [11, 12], the formula is standardly used when judging paediatric electrocardiograms (ECG) e.g. when screening children for congenital long QT syndrome (LQTS). Recently, a proposal was published to use ECG during standing rather than supine position to increase the sensitivity of LQTS screening [13]. Bazett formula was used in the data supporting this proposal.

Nevertheless, the properties of the Bazett formula should also be considered when evaluating paediatric recordings since the erroneously prolonged QTc values at increased heart rate might lead to an increased number of false positive LQTS diagnoses. To investigate this potential problem, we have studied Bazett corrected QTc intervals in a large set of long-term ECG recordings obtained in children during postural provocative manoeuvres [14].

## Methods

### Population and ECG recordings

The investigated population was reported in detail before [14]. In brief, continuous 12-lead ECGs sampled at 1000 Hz were obtained in 345 healthy children and adolescents aged from above 4 to below 15 years (174 girls). Each of the participants underwent a 70-min protocol of postural provocations that consisted of episodes (in the following order) of supine, sitting, standing, supine, standing, sitting, and supine positions, each lasting 10 min. The changes between the body positions were achieved within 20 s, the non-supine positions did not involve any external support. During the investigation, younger children listened to calming age-appropriate stories read by the investigator, others were investigated in an environment free of noise and of other external disturbances.

The source study excluded subjects on interfering drug therapy, cardiac structural congenital abnormalities (including those with a history of cardiac surgery), cardiac conduction abnormality, and (in one case) of a technical recording failure. The study was approved by the Ethics Committee of the University Hospital Brno. The parents or legal guardians of all participants gave informed written consent according to the Helsinki declaration.

In all subjects, the ECG recordings were longer than the 70-min of the investigated protocol since, for practical reasons, the recorders (SEER MC vers 2.0 of GE Healthcare, Milwaukee, WI, USA) were started before and switched off after the postural investigations which were organised in groups of up to 20 participants of similar ages.

### ECG measurements and heart rate correction

As previously described [14], the complete recordings were divided into consecutive 10-s ECG segments and in each segment, heart rate was measured based on the average of all RR intervals.

In each segment, representative median beat was also constructed, and QT interval duration was measured using previously published procedures [15, 16] in the image of the representative beats of all 12 leads superimposed on the same isoelectric axis. As previously described [14], appropriate validation including the measurements including systematic morphological interpretation [17] was employed.

For the purposes of the present investigation, only the 10-s ECG segments obtained during the individual postural positions were considered. To model a population screening strategy in which standard 10-s ECG recordings are obtained, we have not considered the correction for QT/RR hysteresis [18] in the present investigation. Rather, in each of the 10-s ECG segment, QT interval was corrected for the average of the RR intervals measured in the same segment. Three correction formulas were used:

- Bazett correction [5] $$ \mathrm{QTc}=\mathrm{QT}/\sqrt{\mathrm{RR}} $$,
- Fridericia correction [6] $$ \mathrm{QTc}=\mathrm{QT}/\sqrt[3]{\mathrm{RR}} $$,
- Framingham correction [7] QTc = QT + 0.154(1 − RR),

where the QT, RR, and QTc intervals are expressed in seconds.

For each child, previously described individual QT/RR profiles including the QT/RR hysteresis correction was also available from previous investigations [14] but these were only used in this study to ascertain that none of the investigated children had any cardiac repolarisation abnormality.

### Models of long-QT screening

As a small minority of the children were unable to complete the whole protocol, only those children in whom repeated QT and heart rate readings were available during supine, sitting, and standing positions were included into the analyses presented here. For each child, the QTc values according to each of the correction formulas were averaged separately in each of the supine, sitting, and standing positions. Subsequently, for each of the positions, numbers of children in whom the averaged QTc values exceeded 440, 460, and 480 ms were identified and compared between the different correction formulas.

The threshold of 440 ms was selected as an upper borderline upper limit of normality [19], the threshold of 460 ms was selected as an indication of a clear abnormality, and the limit of 480 ms was selected as an definite diagnostic tool of a long QT syndrome [20] (especially if obtained as an average of repeated ECG recordings as was the case in this investigation).

### Individual QT/heart rate profiles

To elucidate the relationship between the correction formulas used in the study and the subject-specific profiles of QT/RR relationship, correlation coefficients were investigated between the averaged RR intervals in separate 10-s ECG segments and the theoretical QTc intervals given as QT/RR^α^ and QT + β(1-RR). These intra-subject correlations were investigated ranging the coefficients α and β between 0 and 1 with the aim of studying the distribution of the coefficients that lead, for each child, to the zero correlation between repeated RR and QTc measurements. This was based on the principle that a successful heart rate correction eliminates the QT dependency on the underlying heart rate [2]. These calculations were made only using the measurements made in ECG segments recorded when the study subjects were in the pre-specified postural positions.

### Statistics

Descriptive data are presented as means ± standard deviation. In addition to the categorical analysis based on upper QTc limits, heart rates and absolute QTc values at different postural positions, and their changes between supine and other positions were statistically summarised and compared between girls and boys. These comparisons were based on two-sample two-tail t-test assuming different variances of compared samples. The intra-subject differences in heart rate and in QTc intervals between postural positions were evaluated using paired two-tail t-test. Correlation between QTc changes and heart rate changes was evaluated using Pearson correlation coefficients. *P*-values below 0.05 were considered statistically significant. The statistical analyses were made using SPSS vers 26 (IBM, Armonk, NY, USA).

## Results

Complete heart rate and QT interval data of supine, sitting, and standing positions were available in 332 children (age range 4 years, 7 months to 14 years, 11 months). The population included 166 girls (aged 10.7 ± 2.7 years) and 166 boys (aged 10.8 ± 2.6 years). The previously reported study of full QT/RR profiles [14] confirmed that none of the children had any electrocardiographic repolarisation abnormality. That is, in each child, the full profile of individual QT/heart rate adaptation was measured and studied, and ECGs at different heart rates were visually reviewed to identify repolarisation pathologies. None of these were found.

### Postural changes

The absolute values of heart rates and of QTc intervals are shown in Table 1; their intra-subject differences associated with the postural changes are shown in Table 2. As expected, heart rate increased when changing the position from supine to standing. The extent of the heart rate change was perhaps less expected since the change from supine to sitting led, on average, to a heart rate increase by 13.4 ± 7.8 bpm, whilst the position change from supine to standing increased heart rate, on average, by 23.5 ± 10.1 bpm.

**Table 1:** Absolute values of ECG measurements

	Girls				Boys			
	Heart rate	QTc_Bazett_	QTc_Fridericia_	QTc_Framingham_	Heart rate	QTc_Bazett_	QTc_Fridericia_	QTc_Framingham_
Supine	78.00 ± 10.42	426.27 ± 15.36	408.3 ± 13.77	408.73 ± 13.01	76.96 ± 10.55	424.32 ± 16.26	407.27 ± 14.05	407.69 ± 13.17
Sitting	92.72 ± 9.55	436.93 ± 14.48	406.47 ± 12.98	405.47 ± 11.69	88.98 ± 10.36	433.00 ± 15.46	405.60 ± 13.44	405.17 ± 12.25
Standing	101.88 ± 10.19	445.9 ± 15.68	408.27 ± 13.57	405.08 ± 11.85	100.01 ± 10.75	443.97 ± 16.07	407.84 ± 13.81	405.13 ± 12.22

For individual postural positions, the table shows heart rate (in beats per minute) and QTc intervals according to the 3 considered correction formulas (in milliseconds). The only significant differences between girls and boys were for the sitting heart rate (*p* = 0.0007) and for the sitting Bazett corrected QTc intervals (*p* = 0.018). Note also that median values (not shown) were practically identical to the presented mean values

**Table 2:** Postural changes of ECG measurements

	Girls				Boys			
	Heart rate	QTc_Bazett_	QTc_Fridericia_	QTc_Framingham_	Heart Rate	QTc_Bazett_	QTc_Fridericia_	QTc_Framingham_
Sitting-supine	14.72 ± 7.85	10.66 ± 8.14	−1.83 ± 4.70	−3.25 ± 5.06	12.02 ± 7.62	8.68 ± 7.90	−1.66 ± 3.65	−2.51 ± 3.65
	< 0.0001	< 0.0001	< 0.0001	< 0.0001	< 0.0001	< 0.0001	< 0.0001	< 0.0001
Standing-supine	23.87 ± 9.84	19.63 ± 11.69	−0.04 ± 7.50	−3.65 ± 7.27	23.06 ± 10.43	19.65 ± 11.22	0.57 ± 5.50	−2.55 ± 5.42
	< 0.0001	< 0.0001	NS	< 0.0001	< 0.0001	< 0.0001	NS	< 0.0001

For the position changes from supine to sitting and supine to standing, the table shows changes in heart rate (in beats per minute) and in QTc intervals according to the considered QTc formulas (in milliseconds). For the positional changes, the table also shows *p*-value (bottom numbers) of the paired t-test comparing the sitting and standing values with the supine values. The only significant differences between girls and boys were for the sitting vs supine heart rates (*p* = 0.0017) and for the sitting vs supine Bazett corrected QTc intervals (*p* = 0.025)

Importantly, while Fridericia and Framingham corrections led only to minimal and clinically clearly unimportant QTc changes (on average by small single milliseconds) from supine to sitting and to standing positions, Bazett corrected QTc intervals increased noticeably by, on average, 9.7 ± 8.1 ms and 19.6 ± 11.4 ms when the position was changed from supine to sitting and to standing, respectively.

**Fig. 1:**
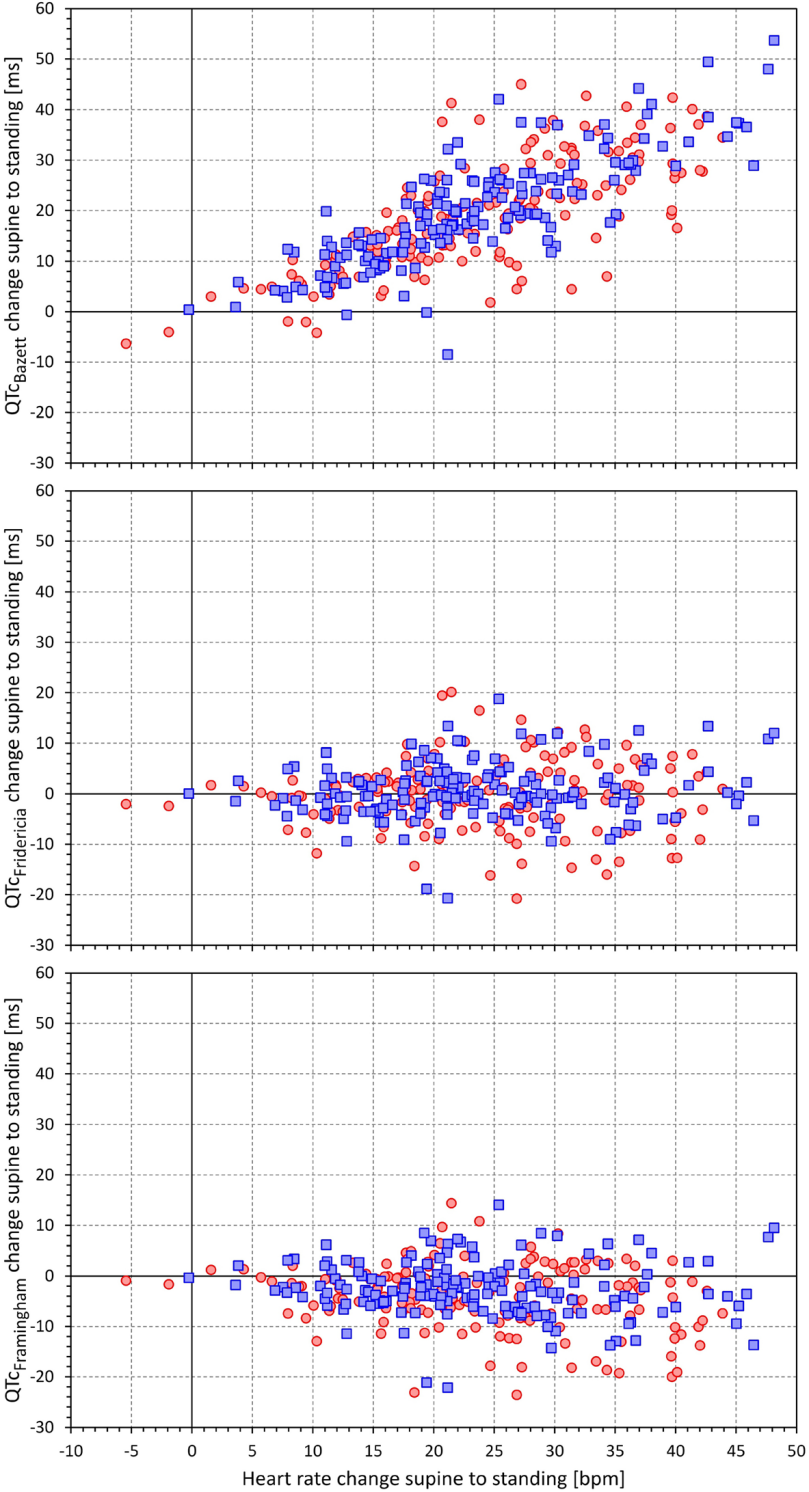
Scatter diagrams between heart rate changes from supine to standing (the same horizontal axis in all panels) and QTc changes reported by the Bazett formula (top panel) Fridericia formula (middle panel) and Framingham formula (bottom panel). In each panel, the red circles correspond to girls, and the blue squares to boys. Note the obvious positive correlation between the heart rate changes and the changes of the Bazett corrected QTc intervals as well as a slight negative correlation between the heart rate changes and the changes of the Framingham corrected QTc intervals

The supine to sitting and supine to standing changes of Bazett corrected QTc intervals were highly significantly correlated to the corresponding changes of heart rate. The corresponding correlation coefficients were r = 0.817 (*p* < 0.00001) and r = 0.764 (p < 0.00001), respectively. No significant correlation was found between the heart rate changes and the changes of Fridericia corrected QTc intervals (correlation coefficients of r = − 0.034, and r = 0.064, for changes to sitting and standing, respectively, both NS). The changes of the Framingham corrected QTc intervals were mildly negatively correlated to the corresponding changes of heart rate with correlation coefficients of r = − 0.182 (*p* = 0.001) and r = − 0.150 (*p* = 0.008) for changes to sitting and standing, respectively. The relationship between the heart rate changes and the QTc changes of the postural changes from supine to standing are shown in Fig. 1.

### Detection of children with a prolonged QTc interval

Using the QTc intervals in supine, Bazett correction identified 51 children (15.36%) as having QTc interval between 440 and 460 ms, and 4 children (1.20%) as having QTc interval between 460 and 480 ms. When QTc intervals in sitting were used, Bazett correction found 118 children (35.54%) as having QTc interval between 440 and 460 ms, 11 children (3.31%) as having QTc interval between 460 and 480 ms, and 1 child (0.30%) as having the QTc interval above 480 ms. When QTc intervals in standing were used, the corresponding numbers of children identified with QTc prolongation increased to 156 (46.99%), 45 (13.55%), and 5 (1.51%), respectively.

Fridericia correction identified only 4 children (1.2%) in supine and 6 children (1.81%) in standing as having QTc interval between 440 and 460 ms. For Framingham correction, the corresponding numbers were 3 children (0.90%) and 1 child (0.30%), respectively. Using these corrections, no children were found to have the QTc interval above 440 ms in sitting recordings.

**Fig. 2:**
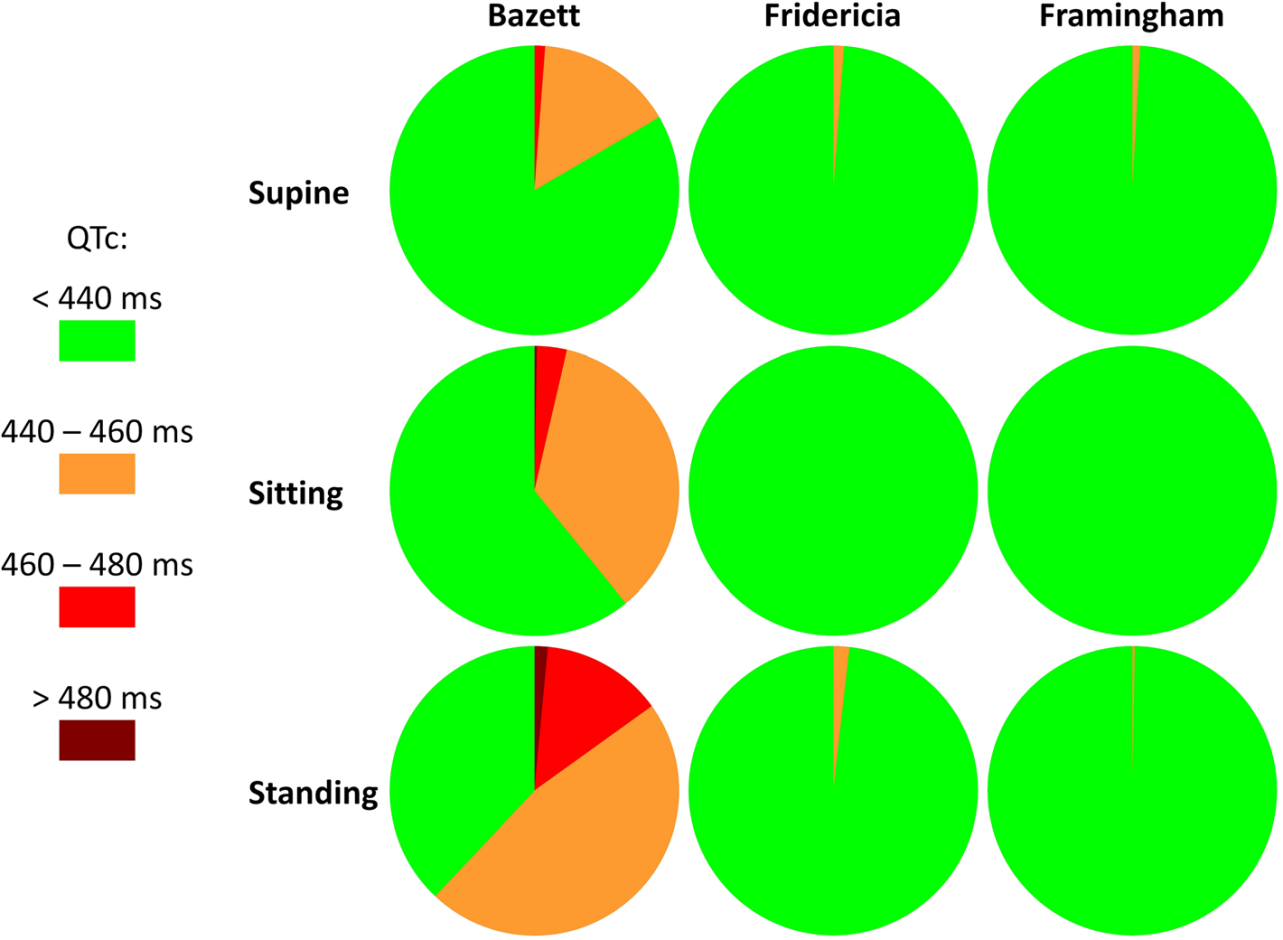
For each of the correction formulas and for each of the postural positions, the corresponding pie chart shows the proportion of children who were found to have the QTc interval below 440 ms (in green), between 440 and 460 ms (in amber), between 460 and 480 ms (in red) and above 480 ms (in dark brown). Note the substantial differences between the Bazett formula and the other two formulas, especially when applied to the interval measurements in the standing ECGs

The summary of these observations is graphically displayed in Fig. 2.

### Subject-specific QT/RR relationships

**Fig. 3:**
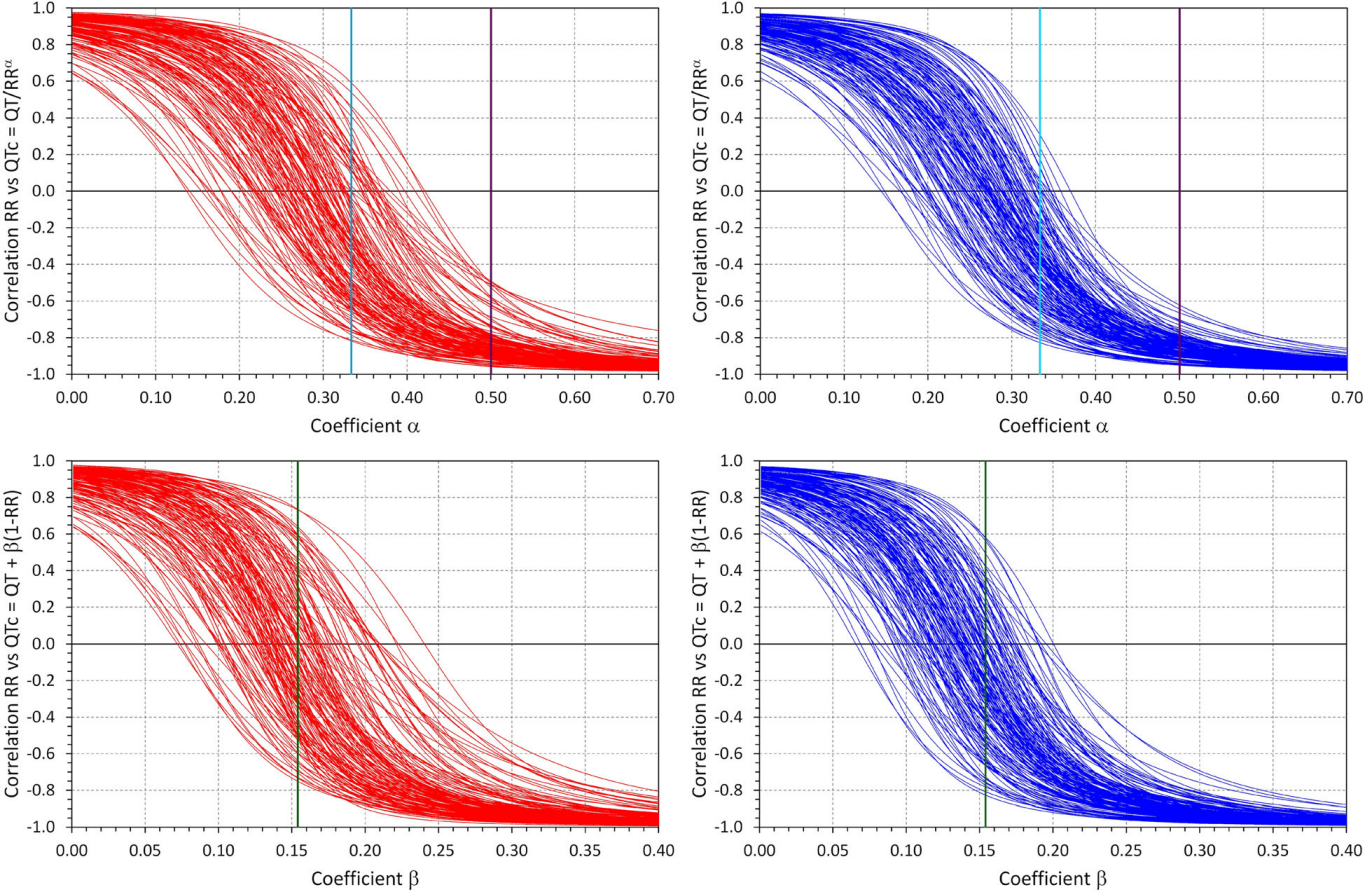
The top panels show the subject-specific correlations between RR intervals and QTc values calculated as QT/RR^α^; the bottom panels show the same for QTc values calculated as QT + β(1-RR), as dependencies on the values of coefficients α (top panels) and β (bottom panels). For each subject, one line is shown and where this line crosses the zero-correlation line, optimum coefficient α or β is found for the given subject, i.e. a coefficient that produces QTc values uncorrelated to the RR values. The panels with red and blue lines show the data of girls and boys, respectively. In the top panels, the light cyan and violet vertical lines show the correction coefficients of the Fridericia and Bazett correction, respectively. In the bottom panels, the dark green vertical lines show the correction coefficient of the Framingham correction

The difference between Bazett formula and Fridericia and Framingham formulas is also shown in the displays of Fig. 3. While the Bazett correction coefficient of 0.5 is well outside the spread of the correction coefficients optimised for the study data in individual subjects, the correction coefficients of the Fridericia formula (0.333) and of the Framingham formula (0.154) are well within the range of the inter-subject spread of the subject-specific optimisations of the log-linear and linear QT/RR regressions.

**Fig. 4:**
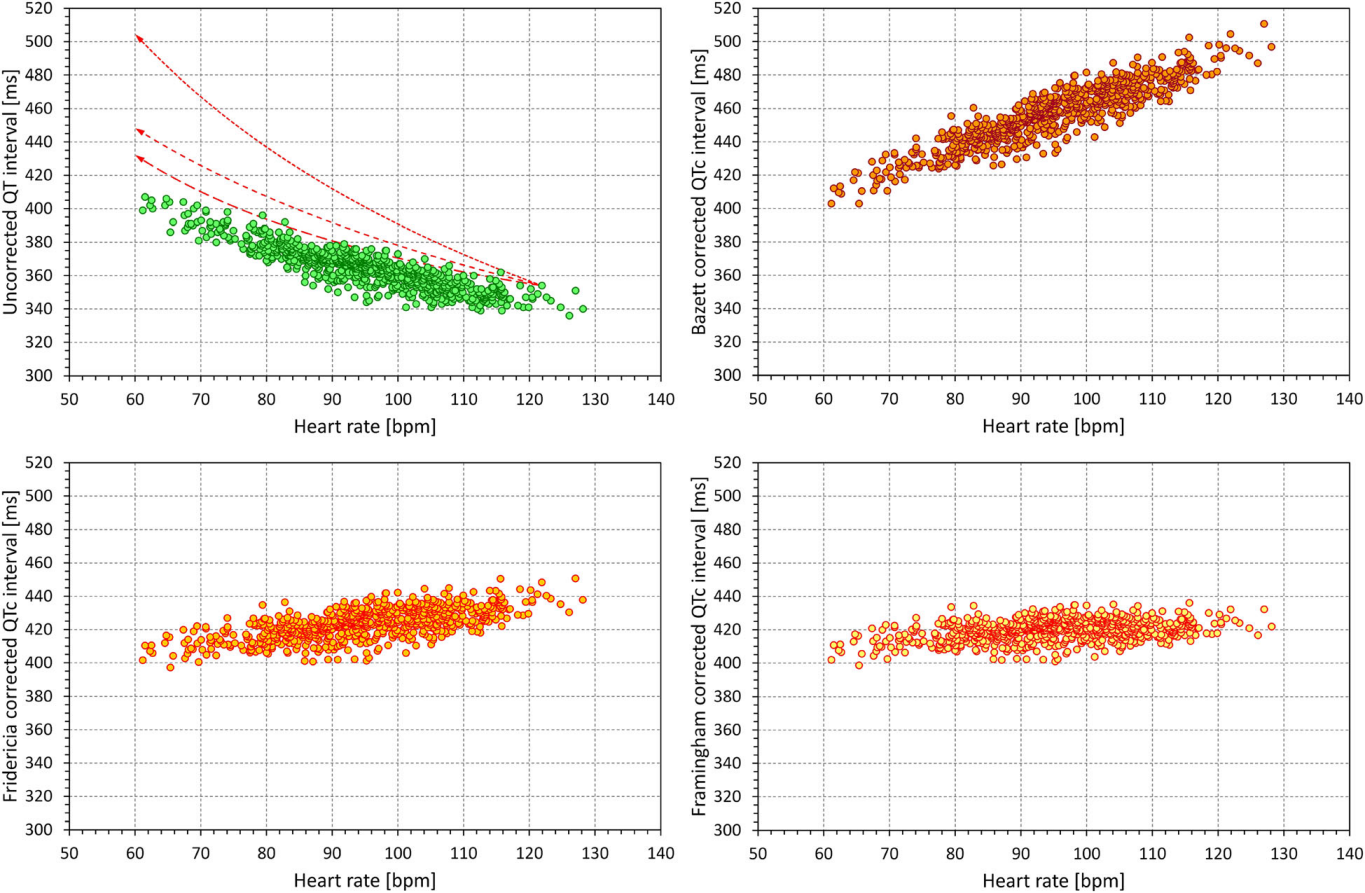
Example of the application of different QTc corrections to QT and heart rate data measured in an 8-year old boy. All the measurements (not only those made during postural provocations) made in the recordings are shown. The top left panel shows a scatter diagram between uncorrected QT intervals and the corresponding 10-s heart rates, the top right, bottom left, and bottom right panels show scatter diagrams between Bazett, Fridericia, and Framingham corrected QTc intervals and the heart rates, respectively. The top left panel shows that in this child, QT interval was adapting to heart rate changes in a rather shallow manner and that the true QTc value in this child was somewhere between 410 and 420 ms. Red arrow in this panel identifies one particular ECG segment in which an uncorrected QT interval of 354 ms was measured at the heart rate of 121.9 bpm. Since the Bazett correction assumes that the QT interval adapts to heart rate along a square root of the RR intervals (short-dash red line in the top left panel) it leads to QTc of 505 ms. Fridericia and Framingham corrections assume different heart rate adaptations (middle-dashed and long-dashed lines in this panel, respectively) and thus lead to QTc of 448 and 432 ms, respectively. Hence in this particular child, Framingham correction is the closest to the true QT/RR profile but that does not mean that the same formula preference would be found with other children. Only Bazett correction overcorrected the QT/RR profile in all children of our study

An example of the comparison of the effects of Bazett correction with the corrections of the other two formulas is also shown in Fig. 4 that shows the scatter diagrams of multiple 10-s QT and heart rate measurements in a recording of an 8-year old healthy boy.

## Discussion

In adult electrocardiography, criticism of Bazett correction has been repeatedly published [11, 21] and some of these studies also referred to problems in children and adolescents [22, 23]. While in adult cardiology, the inaccuracies of Bazett correction might be mitigated by applying it only to recordings with heart rates not far from 60 bpm, this is hardly possible in children. The overestimation of Bazett corrected QTc intervals at higher heart rates thus need to be considered. This is of particular concern if the heart rate is further elevated, e.g. by exercise test or by postural manoeuvres, as was the case in the data that we presented. Therefore, while it is possible to agree with Reynisson et al. [13] that judging ECGs recorded in the standing position increases the sensitivity of LQTS diagnoses, our data show that this increase of sensitivity comes at a very high cost of substantially decreased specificity.

Indeed, all the children included in our study were previously confirmed to be free of QTc interval prolongation based on details of the QT/RR profiles [14]. This fits well with the applications of Fridericia and Framingham corrections that only occasionally identified small number of children with potentially moderate QTc prolongation. On the contrary, Bazett correction identified 16.5% of children as having QTc above 440 ms when supine recordings were used, and this proportion increased to 25.7 and 61.7% (i.e. almost two thirds) when sitting and standing recordings were analysed, respectively. Projecting these results into clinical practice makes it clear that any screening strategy would easily be overwhelmed by a substantial number of false positive cases (these were probably not encountered by Reynisson et al. [13] because of the relatively small number of their control cases).

The Fridericia and Framingham corrections were originally developed by studying QT/heart-rate relationship in large populations [6, 7] while aiming at finding coefficients that would, in these populations, make the QTc values independent of the RR intervals. Bazett correction was not based on any such calculations [5]; indeed, when re-analysing the data available to Captain Bazett, a correction coefficient closer to Fridericia correction is found [24]. More importantly, however, the independence of QTc values of RR values in a population does not imply such an independence in individual subjects. Previous proposals of heart rate corrections were thus influenced by the investigated populations and large number of different formulas have been proposed [22, 25, 26]. Nevertheless, it is now understood that because of the inter-subject differences [3, 27], none of these universal formulas can possibly make the QTc intervals independent of heart rate in each individual. Other problems with the “population-optimised” corrections have also been recognised [28]. This implies that excesses of heart rate are undesirable when making clinical judgements of QTc intervals and that formulas that are close to the “middle” of individual QT/RR profiles are preferable over the Bazett correction that is as far from the spread of the QT/RR profiles in adult subjects [29] as in our population of healthy children.

Superficially, our results might be perceived as contradicting all the studies that previously used Bazett formula in paediatric diagnostics. Of these, valid comparison between different formulas was published by Stramba-Badiale at al [30] finding little differences between different formulas with a slight preference for Bazett correction. Nevertheless, this study reported ECGs in newborns in whom the uncorrected QT values are numerically short and thus subject to different effects of logarithmic transformation [31]. Similar conclusions were reached by Phan et al. [32] but again in little babies and children below 2 years of age. It is not known whether the individuality (that is, the combination of inter-subject differences with intra-subject stability) of QT/RR profiles exists already in newborns and small babies or whether is gradually develops and becomes detectable in school-aged children whom we investigated. Moreover, when considering only ECGs with strictly normal resting heart rates, we found lesser diagnostic differences between the compared formulas (see Fig. 2). The main problem highlighted by our study therefore relates to the elevated heart rates as recently advocated by Reynisson et al. [13].

Our results concerning Fridericia and Framingham formulas are in good agreement with the regulatory suggestions that show that these formulas are preferable to others when studying QTc stability under the conditions of heart rate changes. Superficially, the slight (and clinically clearly unimportant) decreases in QTc at sitting and standing compared to supine might create an impression that these formulas suffer from the opposite problem of the Bazett formula. Nevertheless, this is not justified since Fig. 3 shows clearly that in the majority of the children of our population, both Fridericia and Framingham formulas led to a positive rather than to a negative correlation between QTc intervals and the RR intervals of the underlying heart rate. Detailed analysed of the data show that the minimally negative QTc changes were caused by a combination of the individual QT/RR curvatures (that were not fully reflected in the linear and log-linear regression models) and by the distribution of the heart rate changes in the population. The negative shallow correlations between the heart rate changes and QTc changes reported by the Framingham formula were also caused by the individual QT/RR profiles since in these children, the QT interval did not respond to the RR interval in a strictly linear fashion as expected by the formula. Our data thus suggest that Fridericia formula is marginally preferable to Framingham formula in paediatric applications.

Since the Fridericia formula is now the method of choice when studying the drug-induced LQTS in regulatory analyses of clinical studies [4], we believe that the accuracy of LQTS diagnoses in children would also benefit from using Fridericia formula instead of the Bazett formula which, as we have shown, is likely to lead to an unacceptable number of false positive findings.

### Limitations

Intentionally, we included only healthy children free of any electrocardiographic repolarisation abnormality in this study. That allows us to demonstrate the substantial problem of false positive QTc prolongation linked to the use of Bazett correction, but we are unable to assess the incidence of false negative cases. Since a known proportion of children with congenital LQTS may present with normal QTc interval duration [33] even at heart rate close to 60 bpm (where any heart rate correction is of little importance) it is obvious that LQTS diagnosis cannot be solely based on ECG readings. To model a population screening strategy, we analysed our data only using separate 10-s ECG segments. That prevented us from considering QT/RR hysteresis [18] that is known to influence the accuracy of heart rate correction. The Fridericia and Framingham formulas were selected from a wide spectrum of other published formulas. Other correction possibilities might also eliminate the false positive problem associated with Bazett correction. Nevertheless, in adult data, Fridericia and Framingham formulas appeared to outperform other corrections [9].

## Conclusion

Despite these limitations, our data show that the use of Bazett correction in paediatric studies leads to problematic findings of artificially prolonged QTc intervals, especially if the recent suggestions are followed and if the underlying heart rates are increased by postural changes. Consistent with the diagnosis of acquired LQTS in adult subjects, Fridericia correction formula can be proposed for improved ECG evaluations.

## Data Availability

The data that support the findings of this study are available from the authors upon reasonable request.

## Abbreviations

bpm: Beats per minute
ECG: Electrocardiogram
LQTS: Long QT syndrome
ms: Millisecond
